# Whole-Genome Sequencing of Two Latin American–Mediterranean Extensively Drug-Resistant *Mycobacterium tuberculosis* Clinical Isolates from Medellín, Colombia

**DOI:** 10.1128/genomeA.00192-16

**Published:** 2016-03-31

**Authors:** N. Alvarez, D. Haft, U. A. Hurtado, J. Robledo, F. Rouzaud

**Affiliations:** a Corporación para Investigaciones Biológicas (CIB), Medellín, Colombia; b J. Craig Venter Institute, Rockville, Maryland, USA; c Universidad Pontificia Bolivariana (UPB), Medellín, Colombia; d Equal Opportunity Life Sciences (EQUOLS), Rockville, Maryland, USA

## Abstract

Colombia, with a tuberculosis incidence of 33 cases per 100,000 population, is one of the countries that have reported extensively drug-resistant *Mycobacterium tuberculosis* (XDR-TB). We report the high-quality draft genome sequences of two Latin American–Mediterranean XDR-TB clinical isolates (TBR-152 and TBR-175), comprising 4,303,775 bp and 4,330,115 bp, respectively.

## GENOME ANNOUNCEMENT

Tuberculosis (TB) is a global public health problem with an estimated 9.6 million incident cases in 2014, of which 480,000 were cases of multidrug-resistant TB (MDR-TB) (1). In 2014, TB caused about 1.5 million deaths worldwide. Extensively drug-resistant TB (XDR-TB) has been reported by 105 countries to date, and an estimated 9.7% of patients with MDR-TB have XDR-TB (1). The continued increase and spread of XDR-TB cases is becoming a serious threat to public health worldwide, underscoring the need for better clinical management and development of new drugs (1,2).

The TB Latin American–Mediterranean (LAM) sublineage belongs to lineage 4, Euro-American, one of the seven major phylogeographical *Mycobacterium tuberculosis* lineages that have been described around the world (3,4). Although it has been reported with considerable variation among countries, LAM is predominant in Latin America (5–7). To better understand the molecular mechanisms involved in the extensive drug resistance of *M. tuberculosis* strains from the LAM sublineage, we sequenced the whole genome of two clinical isolates from Medellín, Colombia.

A 24-locus mycobacterial interspersed repetitive-unit–variable-number tandem-repeat (MIRU-VNTR) profile was used to confirm the assignment of isolates TBR-152 and TBR-175 to the *M. tuberculosis* LAM sublineage (8). Phenotypic susceptibility tests to first- and second-line drugs were performed using the Bactec MGIT 960 method. It was determined that TBR-152 and TBR-175 are resistant to isoniazid, rifampin, ofloxacin, moxifloxacin, and amikacin, classifying them as XDR-TB. Genomic DNA was obtained from both isolates by the cetyl-trimethyl-ammoniumbromide method (9). Samples were treated with RNase prior to paired-end library construction with an average insert size target of 800 bp. Whole-genome sequencing was performed at the J. Craig Venter Institute (Rockville, Maryland, USA) on an Illumina MiSeq platform with 250-bp reads and to about 70× coverage. The draft whole-genome sequences comprise 4,303,775 bp and 4,330,115 bp for TBR-152 and TBR-175, respectively. Both genomes display 65% GC content, and *N*_50_ contig lengths were 81,320 bp for TBR-152 and 67,246 bp for TBR-175. A *de novo* assembly was carried out using Celera Assembler (PMID: 18321888), and structural and functional annotation was completed using multiple-ranked sources of evidence, including the TIGRFAMs (PMID: 23197656) and Pfam (PMID: 24288371) protein family databases. The assemblies contain 4,398 and 4,415 protein-coding genes for TBR-152 and TBR-175, respectively, along with 45 tRNAs for each isolate. Mutations associated with resistance to rifampin (S450L in *rpoB*) and isoniazid (S315T in *katG*) were encountered in both genomes. Also, TBR-152 displayed the *rrs* gene A1401G substitution associated with resistance to aminoglycosides, as well as the *gyrA* S91P mutation associated with resistance to fluoroquinolones. Interestingly, TBR-175 displayed no mutation associated with resistance to either fluoroquinolones or aminoglycosides.

This report on LAM sublineage XDR-TB Colombian isolates will provide material for comparative genomic analysis with other XDR-TB genomes circulating in Latin America and in other regions of the world (10–14).

### Nucleotide sequence accession numbers.

These genome projects have been deposited at DDBJ/EMBL/GenBank under the accession numbers JRJQ00000000 for TBR-152 and JRJR00000000 for TBR-175. The versions described in this paper are the first versions, JRJQ01000000 and JRJR01000000.
